# *Lactobacillus Reuteri* Vesicles Regulate Mitochondrial Function of Macrophages to Promote Mucosal and Cutaneous Wound Healing

**DOI:** 10.1002/advs.202309725

**Published:** 2024-04-22

**Authors:** Yuan Chen, Xiaoyao Huang, Anqi Liu, Siyuan Fan, Shiyu Liu, Zihan Li, Xiaoxue Yang, Hao Guo, Meiling Wu, Meng Liu, Peisheng Liu, Fei Fu, Siying Liu, Kun Xuan

**Affiliations:** ^1^ State Key Laboratory of Oral & Maxillofacial Reconstruction and Regeneration and National Clinical Research Center for Oral Diseases and Shaanxi Clinical Research Center for Oral Diseases Department of Preventive Dentistry School of Stomatology The Fourth Military Medical University Xi'an 710032 China; ^2^ State Key Laboratory of Oral & Maxillofacial Reconstruction and Regeneration and National Clinical Research Center for Oral Diseases and Shaanxi Clinical Research Center for Oral Diseases Center for Tissue Engineering School of Stomatology The Fourth Military Medical University Xi'an 710032 China

**Keywords:** extracellular vesicles, *Lactobacillus reuteri* DSM 20016, macrophages, mitochondria, wound healing

## Abstract

The interplay between bacteria and their host influences the homeostasis of the human immune microenvironment, and this reciprocal interaction also affects the process of tissue damage repair. A variety of immunomodulatory commensal bacteria reside in the body, capable of delivering membrane vesicles (MVs) to host cells to regulate the local immune microenvironment. This research revealed, for the initial time, the significant enhancement of mucosal and cutaneous wound healing by MVs secreted by the human commensal *Lactobacillus reuteri* (RMVs) through modulation of the inflammatory environment in wound tissue. Local administration of RMVs reduces the proportion of pro‐inflammatory macrophages in inflamed tissues and mitigates the level of local inflammation, thereby facilitating the healing of oral mucosa and cutaneous wounds. The elevated oxidative stress levels in activated pro‐inflammatory macrophages can be modulated by RMVs, resulting in phenotypic transformation of macrophages. Furthermore, 3‐hydroxypropionaldehyde present in RMVs can decrease the mitochondrial permeability of macrophages and stabilize the mitochondrial membrane potential, thereby promoting the conversion of macrophages to an anti‐inflammatory phenotype. This study pioneers the significance of commensal bacterial MVs in tissue injury repair and presents a novel concept for the repair of tissue damage.

## Graphical abstract

Interactions between bacteria and the host profoundly impact the homeostasis of the human immune microenvironment. In this study, induced alternative activation of macrophages by modulating mitochondrial function, demonstrating significant potential in promoting mucosal and skin wound healing, thus introducing a novel concept for tissue injury repair.

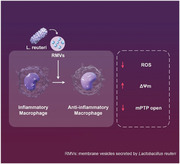

## 1 Introduction

Since birth, human beings embark on a lifelong process colonization by foreign microorganisms.^[^ ^1^ ^]^ Through millennia of evolution, certain bacteria have established symbiotic relationships with their hosts, fostering a mutually beneficial environment.^[^ ^2^, ^3^, ^4^ ^]^ Diverging from opportunistic pathogens that trigger immune responses resulting in tissue damage during infection, several commensal bacterial species exhibit anti‐inflammatory potential.^[^ ^5^, ^6^, ^7^, ^8^ ^]^ The oral mucosal surface harbors a complex microbial community, akin to the extensively studied gut microbiota.^[^ ^9^ ^]^ In line with its gastrointestinal counterpart, the oral mucosal surface microbiota assumes a pivotal role in fostering tissue immunity, repair mechanisms, and defense against pathogens.^[^ ^10^, ^11^, ^12^ ^]^

In comparison to the skin, human oral mucosa characteristic of rapid and scar‐free healing, which is attributed not only to the specific cell types involved in wound repair but also to the significant contribution of certain indigenous microorganisms residing on its surface.^[^ ^13^, ^14^, ^15^ ^]^ According to the World Health Organization, probiotics are “live microorganisms which, if administered in the right amount, benefit the host”.^[^ ^16^, ^17^ ^]^ *Lactobacillus reuteri* (*L. reuteri*), an extensively investigated probiotic strain, can be found colonizing various mucosal surfaces such as the gastrointestinal tract, urinary tract, and oral cavity.^[^ ^18^ ^]^ Significantly, recent research has demonstrated that encapsulation of *L. reuteri* within hydrogel microspheres confers exceptional efficacy against pathogenic bacteria along with anti‐inflammatory properties; thereby promoting wound closure and facilitating tissue regeneration.^[^ ^19^ ^]^

**Scheme 1:**
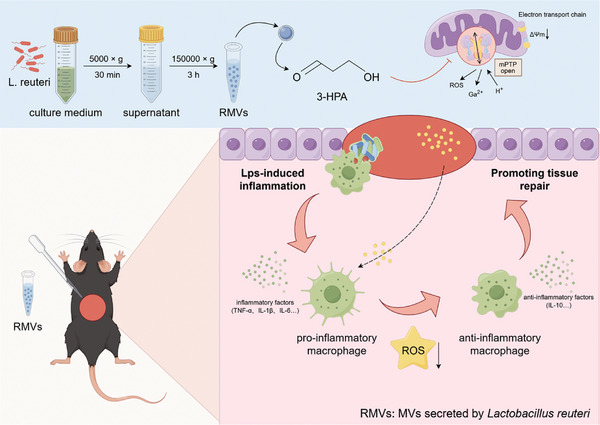
Schematic diagram demonstrates how RMVs shift macrophages to the M2 phenotype, aiding in the wound healing process. (By Figdraw).

Both Commensal Gram‐negative and Gram‐positive bacteria secrete bacterial extracellular vesicles (BEVs) capable of interacting with host cells from various kingdoms. These BEVs deliver their contents to modulate the physiology and function of host cells.^[^ ^20^ ^]^ Inflammation‐including monocytes and macrophages that reside in tissues are essential for controlling tissue healing, regrowth, and scarring.^[^ ^21^ ^]^ Immune cells experience substantial alterations in both appearance and role during tissue damage, aiding in the beginning, continuation, and completion stages of tissue healing.^[^ ^21^, ^22^, ^23^ ^]^ BEVs can penetrate the skin tissue and undergo uptake by tissue‐residing macrophages through mechanisms such as endocytosis, fusion, or active internalization, thereby regulating their phenotypes and influencing the course of local inflammatory responses and tissue regeneration.^[^ ^24^ ^]^ However, the impact of membrane vesicles derived from *L. reuteri* (RMVs) on macrophages and their roles in wound healing remain unexplored. In this study, we initially conducted the preparation of RMVs and evaluated their efficacy in promoting repair of lingual mucosa injury. Subsequently, we assessed the capacity of RMVs to induce alternative activation of macrophages. Mechanistically, our findings demonstrate that 3‐HPA present in RMVs modulates the oxidative stress status of activated macrophages by altering mitochondrial permeability. Finally, we established a mouse skin defect model to validate the pro‐healing effect of RMVs containing 3‐HPA. In conclusion, our findings suggest that RMVs have the potential to mitigate oxidative stress in macrophages, thereby facilitating their transition toward an anti‐inflammatory phenotype, which is conducive to expedited tissue regeneration (**Scheme** **1**).

## 2 Results

### 2.1 Physical Characterization of RMVs

**Figure 1:**
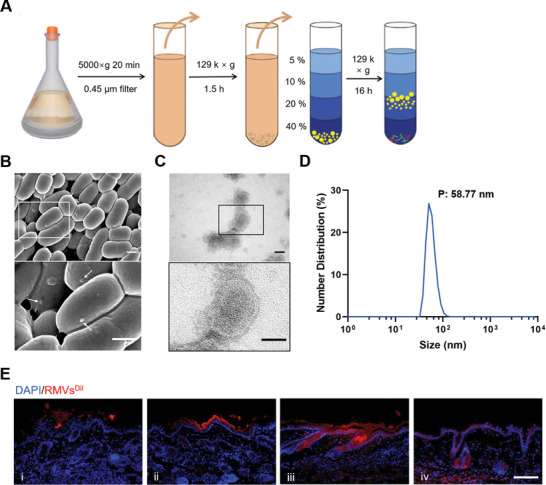
Characterization of RMVs. *Lactobacillus reuteri* DSM 20 016 biofilm development after 48 h of incubation. A) RMVs isolation procedure. B) SEM image showing biofilm with attached vesicles on cells (indicated by arrows). Scale bar, 500 nm. C) TEM showing the bilayer structure of RMVs. Scale bar, 50 nm. The DLS distribution of RMVs particle size is shown in D). E) Representative immunofluorescence staining images of vesicles taken up by the skin over time. i: 6, ii: 12, iii: 24, and iv: 48 h. Scale bar, 100 µm.

RMVs were collected by ultracentrifugation of cell‐free culture broth (**Figure** **1**A) and characterized by dynamic light scattering analysis (DLS), scanning electron microscopy (SEM), and transmission electron microscopy (TEM). The surface of *L. reuteri* is characterized by the presence of prominent round protrusions, referred to as RMVs (Figure 1B). The morphology of RMVs seemed to be enclosed by a spherical membrane (Figure 1C). Based on the results of DLS, the average size of RMVs was ≈60 nm (Figure 1D), which is consistent with previous studies reported in scientific literature. Relevant vesicle uptake experiments were subsequently conducted. After topical application of red fluorescently labeled RMVs to the skin surface of mice, it was observed that some RMVs had penetrated the hair follicles at 24 h, with a substantial number of RMVs localized in the dermal cells surrounding the hair follicles at 48 h (Figure 1E).

### 2.2 RMVs Reduce the Level of Inflammation and Accelerate the Healing of Tongue Ulcers

**Figure 2:**
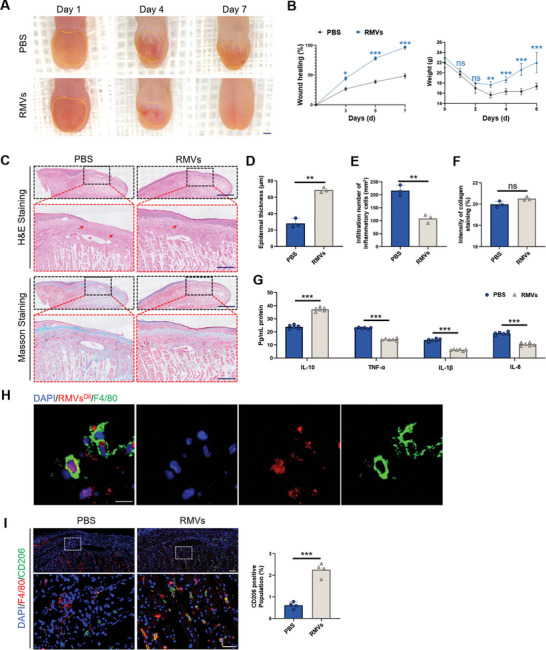
RMVs accelerated the healing of oral mucosal wounds. A) Images showing wounds treated with PBS and RMVs on days 4 and 7 after being wounded. Scale bar, 1 mm. B) The mice's weight and the speed of wound healing at specified intervals. *n* = 6 per group. C) Representative images of H&E staining and Masson's trichrome staining of wound sections were taken 7 days after wound was created. Single arrows indicate regions of inflammatory infiltration. Scale bar, 1 mm in low‐magnification pictures, 200 µm in high‐magnification images. D) Quantification of epidermal thickness. *n* = 3 per group. E) Infiltration number of inflammatory cells. *n* = 3 per group. F) Intensity of collagen staining of newly formed oral mucosal tissue. *n* = 3 per group. G) ELISA was used to analyze the expression of cytokines in wounds of the oral mucosal. *n* = 6 per group. H) In vivo, F4/80‐positive macrophages (green) were observed engulfing RMVs (red) through immunofluorescence staining. Scale bar, 20 µm. I) Representative images and quantification of the CD206 expression (green) in the tongue tissue. Scale bar, 50 µm in low‐magnification images, 20 µm in high‐magnification images. *n* = 4 per group. The data is presented as the mean ± SD. ^**^ *P* < 0.01; ^***^ *P* < 0.001; ns, not significant. RMVs^Dil^, RMVs labeled with Dil.

To investigate whether RMVs can provide therapeutic benefits to oral mucosal wounds, a 3 mm diameter filter paper soaked in glacial acetic was applied to the dorsal tongue of each mouse for 30 s to create a chemical injury wound.^[^ ^25^ ^]^ The mice were separated into two groups: one receiving PBS treatment (referred to as the PBS group) and the other receiving RMVs (5ug in 100 uL PBS) treatment (referred to as the RMVs group). As depicted in **Figure** **2**A, the procession of wound healing was observed by capturing images using a digital camera. In the PBS group, the swelling of the wound sites was obvious on day 4. By day 7, epithelial integrity had not been restored. However, the integrity of tongue mucosa epithelium in RMVs group was completely recovered by day 7, and the degree of tissue edema was also reduced (Figure 2B).

In order to evaluate the process of wound healing, a set of pathological tests were performed, involving the use of hematoxylin and eosin (H&E) staining along with Masson's trichrome staining. As depicted in Figure 2C, H&E staining on day 7 revealed that a significant presence of inflammatory cells were observed in the PBS group, indicating uncontrolled inflammation. By day 7, there were few inflammatory cells left in the RMVs group (Figure 2E), indicating that RMVs have anti‐inflammatory properties. Furthermore, there was scarce evidence of new epidermal tissue formation in the PBS group; however, regenerated epidermis was clear evident in response to RMVs treatment (Figure 2D), highlighting its potential for accelerating wound healing. Moreover, Masson's trichrome staining showed no significant difference between the two groups (Figure 2F).

Relevant inflammatory factors in tongue tissue were subsequently assessed. In comparison to the PBS group, the RMVs group exhibited notable rise in anti‐inflammatory elements like interleukin‐10 (IL‐10) in tongue tissue, along with a reduction in pro‐inflammatory factors such as tumor necrosis factor α (TNF‐α), interleukin‐1β (IL‐1β), and interleukin‐6 (IL‐6) (Figure 2G). The results suggest that RMVs exhibit a pronounced anti‐inflammatory effect on tongue tissue. Furthermore, RMVs were fluorescently labeled to observe their movements after being applied to the wound. After 8 h of treatment with RMVs after wound formation, RMVs were co‐localized with F4/80^+^ cells, indicating that they were engulfed by macrophages in the tissue (Figure 2H). As RMVs were likely to target macrophage, our subsequent investigation focused on the impact of RMVs on these cells. Given the critical role that anti‐inflammatory macrophages play in tissue repair processes, we employed immunofluorescent staining to analyze the expression levels of CD206, a recognized marker for identifying anti‐inflammatory macrophages. The findings indicated a notable rise in the percentage of CD206^+^ macrophages near the wound in the RMVs group, hinting at the potential of RMVs to facilitate the shift of macrophages toward M2‐like phenotypes (Figure 2I). These findings demonstrated that the efficacy of RMVs in treating tongue ulcers, accompanied by an upregulation of M2‐like macrophage expression in the RMVs‐treated group. These results suggest that RMVs may modulate macrophage phenotype to alleviate tongue ulcers.

### 2.3 The Internalization of RMVs by Macrophages can Elicit Polarization toward M2‐Like Phenotypes In Vitro

**Figure 3:**
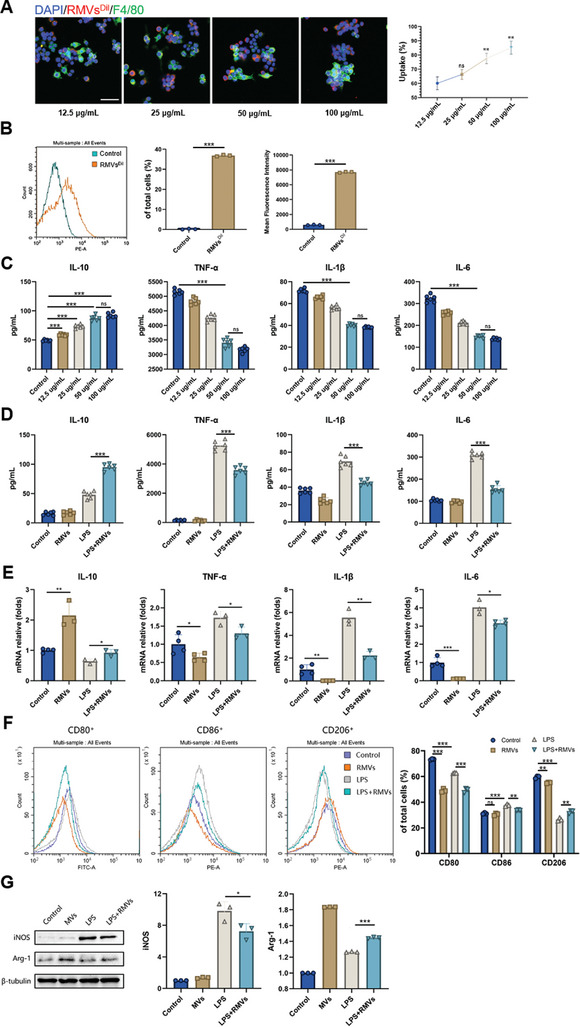
RMVs regulated macrophage function in vitro. A) The immunofluorescence images and quantification of concentration‐dependent uptake of RMVs (red) by RAW 264.7 cells (green). Scale bar, 50 µm. B) Representative flow cytometry histogram and quantification of the distribution of RAW 264.7 cells engulfing RMVs^Dil^. C,D) Cytokines expression in cell supernatant was analyzed by ELISA. *n* = 6 per group. E) RAW 264.7 cells were treated with varying concentrations of RMVs and the cytokine gene expression levels were analyzed using qPCR. *n* = 3 per group. F) Flow cytometry histograms and quantification of CD80, CD86, and CD206 expression levels in RAW 264.7 cells. G) Western blot analysis of the phenotypic markers in RAW 264.7 cells. Proinflammatory macrophage marker, iNOS; anti‐inflammatory macrophage markers, Arg‐1. Data shown as mean ± SD. ^*^ *P* < 0.05; ^**^ *P* < 0.01; ^***^ *P* < 0.001; ns, not significant.

Since we noticed that macrophages were probably the main cells affected by RMVs in a living organism and there was higher number of CD206^+^ cells, our following study concentrated on analyzing how RMVs influenced macrophage polarization in a controlled environment. Macrophages display notable diversity, with the ability to shift toward various functional phenotypes based on the pathogens and cytokines in their surroundings.^[^ ^21^ ^]^ First, we labeled RMVs with a red fluorescent dye (Dil) and then examined the phagocytosis of macrophages. Confocal laser scanning microscopy analysis revealed that after 3 h of incubation with labeled RMVs in the macrophage culture medium, the RMVs were internalized and transported to the perinuclear area of the macrophages (**Figure** **3**A). Simultaneously, we conducted quantitative flow cytometry analysis on DiL‐labeled RMVs‐engulfing cells, revealing that approximately one‐third of macrophages phagocytosed RMVs (Figure 3B).

Next, we employed lipopolysaccharide (LPS) as a straightforward in vitro technique to replicate the inflammatory microenvironment experienced by macrophages. Following LPS stimulation, different concentrations of RMVs were administered to the stimulated macrophages, while unstimulated macrophages served as the control group. The levels of various inflammatory factors in the cell supernatant were assessed using ELISA and found that RMVs could alleviate the elevated levels of pro‐inflammatory factors induced by LPS. It is noteworthy that RMVs can promote the secretion of anti‐inflammatory cytokine (IL‐10) in LPS‐stimulated macrophages, but RMVs does not affect the expression level of inflammatory cytokines in macrophages not activated by LPS (Figure 3C,D). The impact of RMVs varies based on concentration, reaching its peak effectiveness within a range of 50–100 µg ml^−1^. Neither excessively high nor excessively low concentrations yield favorable outcomes (Figure 3C). Qt‐PCR results also proved that RMVs could decrease the mRNA expression of pro‐inflammatory factors and promote the mRNA expression of anti‐inflammatory factors in LPS‐stimulated macrophages (Figure 3E).

Then we labeled CD80, CD86, and CD206 on the surface of macrophages treated with different reagents and detected the cell clusters by flow cytometry. CD80 and CD86 are the surface markers of M1 macrophages, while CD206 is the surface marker of M2 macrophages. LPS promotes the classical activation pathway of macrophages, and the presence of RMV reverses this process (Figure 3F). Meanwhile, protein expression levels of iNOS and Arg‐1 were detected by western blots. The results showed that RMVs could inhibit the increase of iNOS and decrease of Arg‐1 in macrophages induced by LPS (Figure 3G–I). Hence, the results indicate that RMVs help in directing macrophages toward the M2 phenotype, which in turn reduces inflammation and enhanced the process of wound healing.

### 2.4 RMVs Promote Skin Wound Healing In Vivo by Promoting Alternative Activation of Macrophages

**Figure 4:**
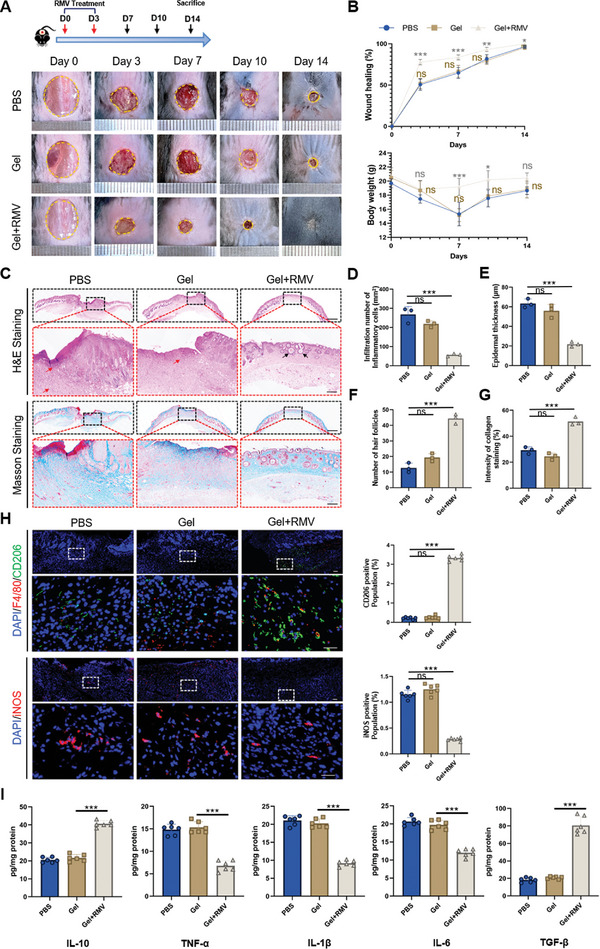
RMVs accelerated the healing of cutaneous wound healing by regulating macrophage phenotype in vivo. A) Representative images of skin injuries in different groups at different time points during the wound healing procedure. B) Quantification of the wound healing rate and the body weight. Each mouse was administered 200 µg of RMVs or 50 µg of 3‐HPA on Day0 and Day7, respectively. C) Representative images of the H&E staining and the Masson staining of the skin samples. The red arrows indicate areas of inflammatory infiltration, while the black arrows highlight nascent hair follicle structures. Scale bar,1 mm in low‐magnification images, 200 µm in high‐magnification images. D) Infiltration number of inflammatory cells. *n* = 3 per group. E) Quantification of epidermal thickness. *n* = 3 per group. F) Quantification of the hair follicles. *n* = 3 per group. G) Collagenvolume fraction of newly formed skin tissue. *n* = 3 per group. H) Representative images and quantification of the CD206 expression in the skin tissue. Scale bar, 50 µm. *n* = 6 per group. I) Cytokines expression and the level of transforming growth factor in cutaneous wounds was analyzed by ELISA. *n* = 6 per group. Data shown as mean ± SD. ^***^ *P* < 0.001; ns, not significant.

We further substantiated the impact of RMVs using a mouse model with a skin defect. There are notable disparities in the healing process between skin and mucosa, including variances in the speed and extent of inflammatory cell infiltration. In the case of adult skin, macrophages are mobilized to the site of injury, where they release various growth factors that stimulate the production of collagen and formation of fibrils.^[^ ^26^ ^]^ To assess the effectiveness of 3‐HPA in promoting wound healing, a comprehensive model for full‐thickness cutaneous wounds was established. For in vivo testing purposes, mice were divided into three groups: PBS treatment (PBS group), hydrogel treatment (Gel group) and RMV hydrogel treatment (Gel+RMV group). As shown in **Figure** **4**A, the Gel+RMV group healed the fastest among the three groups. The body weight of mice in Gel+RMV group did not decrease significantly, while the body weight of mice in the other two groups decreased significantly and returned to normal after nearly two weeks (Figure 4B). The results from H&E and Masson staining indicated a significant improvement in cutaneous wound healing with RMVs compared to both PBS or Gel treatments (Figure 4C). The Gel+RMV exhibited reduced scar size, decreased presence of inflammatory cells, improved integration of skin tissue with regenerated epithelium including hair follicles formation along with better organization and deposition of collagen fibers (Figure 4D–G). The results indicate that using RMVs can speed up the healing of skin wounds and improve the quality of skin regeneration. Subsequently, immunofluorescent staining was performed to assess CD206 and iNOS expression levels (Figure 4H), revealing a notably higher number of CD206^+^ cells within the Gel+RMV group when compared to the other two treatment groups. Simultaneously, there was a notable decrease in the quantity of iNOS^+^ cells indicating the proinflammatory characteristics in the Gel+RMV group in comparison to the remaining two groups. The alterations in the levels of inflammatory factors were also consistent with this finding (Figure 4I). These results suggested that RMVs could facilitate the conversion of pro‐inflammatory macrophages into their anti‐inflammatory counterparts, thereby accelerating the healing of skin wounds.

### 2.5 3‐HPA in RMVs can Relieve the Oxidative Stress State of Activated Macrophages

**Figure 5:**
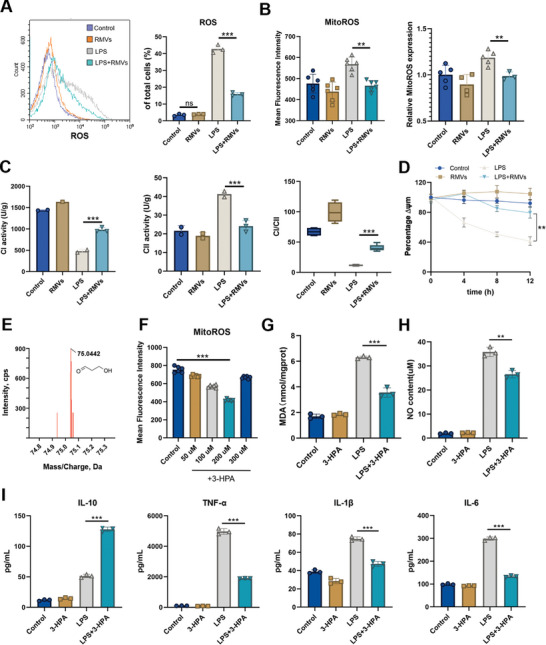
3‐HPA in RMVs relieved the oxidative stress state of activated macrophages. A) Flow cytometry histograms and quantification of expression levels of ROS in RAW 264.7 cells. B) Mitochondrial ROS levels in cells treated with LPS and RMVs. *n* = 6 per group. C) Mitochondrial complex I (CI) and complex II (CII) activities and their ratio CI/CII. *n* = 3 per group. D) The level of mitochondrial membrane potential change. *n* = 3 per group. E) Representative LC‐MS plot of RMVs. F) Mitochondrial ROS levels in macrophages following the addition of 3‐HPA to the medium. *n* = 6 per group. G,H) The levels of MDA and NO. These reflect the cellular oxidative damage. *n* = 3 per group. I) Levels of cytokines secreted by RAW 264.7 cells after the addition of 3‐HPA. *n* = 3 per group. Data shown as mean ± SD. ^**^ *P* < 0.01; ^***^ *P* < 0.001.

During wound healing, macrophages first take on a pro‐inflammatory phenotype and promptly react to infection and damage. After the danger has subsided, the macrophages develop an anti‐inflammatory phenotype respond to facilitate healing and recovery. Several pieces of evidence suggest a strong connection between mitochondrial activity and the production of reactive oxygen species (ROS) during this phenotypic transition. To address the contributions of RMVs in polarization of macrophages, ROS were measured in RAW 264.7 cells. As expected, LPS significantly increased ROS levels in RAW 264.7 cells, including mitochondrial ROS levels (**Figure** **5**A,B). Notably, RMVs appears to have an inhibitory effect on the increase in ROS levels within cells. The addition of RMVs tended to normalize ROS content of LPS‐affected macrophages. The electron transport chain (ETC) is a major component of mitochondrial metabolism and comprises two electron carriers (coenzyme Q and cytochrome c) and four respiratory complexes (CI–CIV). We found that LPS decreased the activity of CI while increasing the activity of CII. RMVs could reverse this process and restore CI and CII activity (Figure 5C). At the same time, changes in mitochondrial complex activity cause changes in mitochondrial membrane potential. However, RMVs could inhibit the changes of mitochondrial membrane potential induced by LPS (Figure 5D).

Reuterin, also known as 3‐hydroxypropionaldehyde (3‐HPA), is primarily produced by *L. reuteri*. 3‐HPA is an intermediate in the metabolism pf glycerol to 1,3‐propanediol. Previous studies have shown that 3‐HPA can change the state of cellular oxidative stress, so we hypothesized that RMVs might function through 3‐HPA. The RMVs samples were analyzed by liquid chromatography‐mass spectrometry and the results showed that 3‐HPA was present in RMVs (Figure 5E). 3‐HPA was able to decrease the levels of mitochondrial ROS in RAW 264.7 cells with a dosage below 200 um (Figure 5F). At the same time, the levels of MDA and NO in the cells were detected, which showed that 3‐HPA could alleviate the oxidative stress state of the cells (Figure 5G,H). Next, we verified the effect of 3‐HPA on the function of RAW 264.7 cells by measuring the content of cytokines by ELISA. Similar to previous findings with RMVs, 3‐HPA was found to elevate the production of anti‐inflammatory factor (IL‐10) while reducing the levels of pro‐inflammatory factors including TNF‐α, IL‐1β, and IL‐6 released by macrophages (Figure 5I). Collectively, the presence of 3‐HPA in RMVs may decrease the level of oxidative stress in macrophages and facilitate their transition to an anti‐inflammatory state.

### 2.6 3‐HPA may Affect the Oxidative Stress of Macrophages by Changing Mitochondrial Permeability

**Figure 6:**
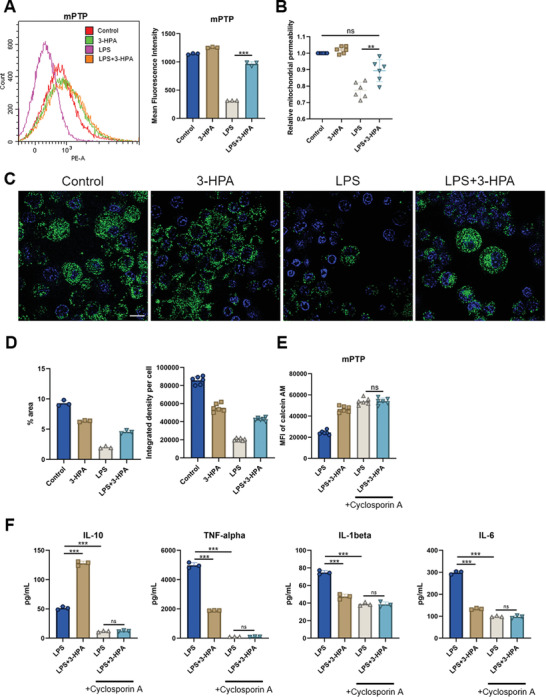
Mitochondrial permeability transition is crucial for the functionality of 3‐HPA. A) Flow cytometry histogram and quantification of mitochondrial permeability in RAW 264.7 cells after addition of 3‐HPA. B) Statistics of relative mitochondrial permeability in RAW 264.7 cells detected by fluorescent microplate reader. *n* = 6 per group. C) Representative fluorescence images of mitochondrial permeability in RAW 264.7 cells. Scale bars, 10 µm. D) Statistics of fluorescence area percentage and integrated density of RAW 264.7 cells. *n* = 6 per group. E) Mitochondrial permeability after addition of cyclosporin A, an inhibitor of cyclophilin D (CypD). F) Cytokines expression in cell supernatant was analyzed by ELISA. *n* = 3 per group. ^**^ *P* < 0.01; ^***^ *P* < 0.001; ns, not significant.

Continuous and extensive activation of the high‐conductance mitochondrial permeability transition pore (mPTP) affects the function of mitochondria, especially the respiratory chain, leading to the breakdown of respiratory super complexes.^[^ ^27^, ^28^ ^]^ The resulting impairment of electron flow leads to an increase in ROS production, which soon exceeds the physiological level associated with ROS signaling. Flow cytometry analysis of mitochondrial permeability in RAW 264.7 cells showed that LPS significantly increased mitochondrial permeability (**Figure** **6**A). This result is consistent with previous ROS assays showing that LPS can open mPTP and promote ROS production. After addition of 3‐HPA, mitochondrial permeability returned to normal. This phenomenon was also proved by the results of immunofluorescence, and the fluorescence intensity was significantly reduced in the LPS group, indicating increased mitochondrial permeability (Figure 6B). However, there was no significant difference in fluorescence intensity between the LPS+3‐HPA group and the control group, indicating that 3‐HPA had an inhibitory effect on the opening of mPTP induced by LPS (Figure 6B). The subcellular structural level can be visualized through super‐resolution fluorescence microscopy (SRM) for the visualization of fluorescent labeling. The changes of mitochondrial fluorescence expression were clearly observed by SRM. 3‐HPA alone did not significantly affect the fluorescence expression in macrophage mitochondria, whereas LPS induced mitochondrial permeability and resulted in quenching of mitochondrial fluorescence (Figure 6C,D). However, 3‐HPA reversed the impact of LPS on mitochondrial permeability in macrophages.

Next, we further investigated the importance of mPTP in the process of 3‐HPA affecting macrophage function. The chaperone cyclophilin D (CypD) is the most effective enhancer of mPTP, and Cyclosporin A (CsA) can prevent mPTP opening by attaching to CypD and causing it to separate from ATP synthase. After adding the CsA, mitochondrial permeability was reduced in the LPS group (Figure 6E). This suggests that CypD is important for the elevation of mitochondrial permeability caused by LPS. Compared with the LPS+3‐HPA group without adding CsA, the mitochondrial permeability of LPS+3‐HPA group with adding CsA did not change significantly. It is possible that 3‐HPA inhibits LPS‐induced mitochondrial permeability by inhibiting the activity of CypD. Furthermore, CsA exhibited a mitigating effect on the pro‐inflammatory response induced by LPS, while the inhibitory impact of 3‐HPA on LPS was nullified (Figure 6F). Therefore, CypD may serve as a potential target for 3‐HPA, necessitating further investigations to ascertain its direct or indirect impact.

### 2.7 3‐HPA Promotes Skin Wound Healing In Vivo by Promoting Alternative Activation of Macrophages

**Figure 7:**
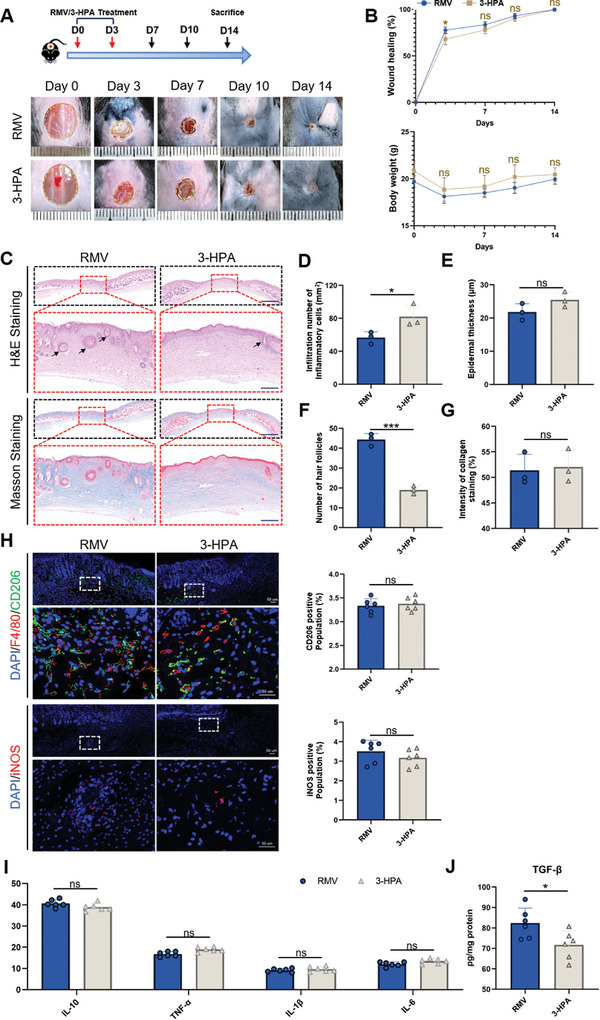
3‐HPA promoted cutaneous wound healing in vivo by promoting alternative conversation of macrophages. A) Representative images of skin injuries in different groups at different time points during the wound healing procedure. B) Quantification of the wound healing rate and the body weight. Each mouse was administered 200 µg of RMVs or 50 µg of 3‐HPA on Day0 and Day7, respectively. C) Representative images of the H&E staining and the Masson staining of the skin samples. The red arrows indicate areas of inflammatory infiltration, while the black arrows highlight nascent hair follicle structures. Scale bar,1 mm in low‐magnification images, 200 µm in high‐magnification images. D) Infiltration number of inflammatory cells. *n = *3 per group. E) Quantification of epidermal thickness. *n = *3 per group. F) Quantification of the hair follicles. *n = *3 per group. G) Collagenvolume fraction of newly formed skin tissue. *n = *3 per group. H) Representative images and quantification of the CD206 expression in the skin tissue. Scale bar, 50 µm. *n = *6 per group. I) Cytokines expression in cutaneous wounds was analyzed by ELISA. *n = *6 per group. J) The level of transforming growth factor‐beta in cutaneous wounds was analyzed by ELISA. *n = *6 per group. Data shown as mean ± SD. ^***^ *P* < 0.001; ns, not significant.

We further established a mouse full‐thickness cutaneous wound model to verify whether 3‐HPA could promote healing by modulating macrophage phenotype. Two groups of mice were separated for testing: RMV hydrogel treatment (Gel+RMV group), and 3‐HPA hydrogel treatment (Gel+3‐HPA group). As shown in **Figure** **7**A, the skin healing speed of Gel+RMV group and Gel+3‐HPA group was basically the same, and the skin healing speed of Gel+RMV group was slightly faster than that of Gel+3‐HPA group within a week. There was significantly decrease in the body weight of mice in either the Gel+RMV or Gel+3‐HPA group (Figure 7B). H&E and Masson staining showed that both RMVs and 3‐HPA had formed continuous epithelial structures at two weeks, but Gel+RMVs group had formed more follicles (Figure 7C,F). There were no significant differences in the degree of inflammatory infiltration, epidermal thickness, and collagen deposition fraction between the two groups (Figures 7D and 6E,G). These data demonstrated comparable efficacy between 3‐HPA alone and RMVs in promoting cutaneous wound healing in mice. Subsequently, we assessed the levels of CD206 and iNOS expression through immunofluorescent staining (Figure 7H). The results indicate that 3‐HPA has a similar ability to promote macrophage phenotype switching as RMVs. The alterations in the levels of inflammatory factors were also consistent with this finding (Figure 7I). Mice treated with RMVs showed notable increased in transforming growth factor β (TGF‐β) expression in the skin compared to those treated with 3‐HPA alone, suggesting that RMVs may encompass extra components that facilitate TGF‐β expression. The correlation component analysis needs to be further studied. In conclusion, the 3‐HPA in RMVs can induce macrophage phenotype switching and exert a pro‐healing effect similar to that of RMVs as a whole.

## 3 Discussion

Impaired wound healing significantly impacts individuals' physical and psychological well‐being.^[^ ^29^ ^]^ In recent years, the pivotal role of microorganisms in host physiological and pathological processes has garnered considerable attention.^[^ ^30^, ^31^ ^]^ Numerous studies have demonstrated that beneficial microorganisms possess the ability to modulate detrimental inflammatory responses and facilitate tissue regeneration. *L. reuteri* is a extensively investigated probiotic strain, which can be found colonizing various mucosal surfaces such as the gastrointestinal tract, urinary tract, and oral cavity.^[^ ^18^ ^]^ Research has shown that exopolysaccharides (EPS) derived from *L. reuteri* strain DSM 17 938 (EPS‐DSM17938) can prevent enterotoxigenic *Escherichia coli* (ETEC) from attaching to IPEC‐1 cells and ETEC‐induced gene expression of proinflammatory cytokine IL‐1β and IL‐6.^[^ ^32^ ^]^ Moreover, the secretion of reuterin by *L. reuteri* has demonstrated its ability to modulate redox balance and effectively inhibit the proliferation and survival of colon cancer cells.^[^ ^33^ ^]^ It is worth noting that in addition to the bacteria themselves, some polysaccharides and metabolites secreted by the microbe possess regulatory capabilities over host cell functions. Various types of bacteria, including both commensal Gram‐negative and Gram‐positive bacteria, have the ability to create BEVs. These BEVs have the ability to interact with host cells and affect their physiology and function by delivering their contents and cargo across different kingdoms.^[^ ^20^ ^]^ In recent years, a large number of studies have focused on the role of BEVs in intestinal homeostasis. The oral cavity and skin are significant sites for microbial accumulation, but the effects of oral microorganisms on the physiological and pathological processes of the host are still unclear. In this study, we discovered that BEVs secreted by *L. reuteri* possess the ability to counteract the mitochondrial permeability transition induced by LPS in macrophages, modulate macrophage phenotype, and influence the inflammatory process, thereby facilitating wound healing of tongue mucosa and skin.

Tissue‐resident macrophages are essential for controlling tissue healing, renewal, and scarring.^[^ ^21^ ^]^ Macrophages serve as an important source of chemokines, TNF‐α, and other inflammatory mediators that initiate the initial cellular response after injury.^[^ ^34^, ^35^ ^]^ Afterward, as the initial inflammatory stage resolves, the main group of macrophages shifts toward a healing state that is marked by high levels of growth factor secretion, including platelet‐derived growth factor (PDGF), TGF‐β, insulin‐like growth factor 1 (IGF‐1), and vascular endothelial growth factor α (VEGF‐α).^[^ ^36^, ^37^ ^]^ These factors promote cellular proliferation and angiogenesis. Furthermore, macrophages that exhibit anti‐inflammatory characteristics are activated by IL‐10 and other inhibitory factors to release different anti‐inflammatory substances like IL‐10 and TGF‐β, which are crucial in suppressing the immune system.^[^ ^21^ ^]^ Therefore, precise regulation is essential for coordinating distinct macrophage phenotypes at different stages of tissue repair. In this work, our findings demonstrate that RMVs possess the ability to inhibit LPS‐induced macrophage polarization toward a pro‐inflammatory phenotype, promote their polarization toward an anti‐inflammatory phenotype, and reduce inflammation levels in vitro. Furthermore, in mouse models of lingual mucosa and skin defects, we show that RMVs can modulate macrophage phenotype switching to regulate inflammation and promote tissue healing.

Mitochondria are essential for controlling inflammatory reactions.^[^ ^38^ ^]^ The metabolic reprogramming induced by Toll‐like receptor 4 (TLR4) activation through the Gram‐negative bacterial cell‐wall component LPS leads to an increase in glycolysis. In this process, macrophages undergo a transformation in their mitochondrial function, shifting from ATP production to the generation of ROS dependent on succinate. This change allows glycolysis to take over the role of ATP generation, enabling mitochondria to maintain a high membrane potential.^[^ ^39^ ^]^ ETC, an essential component of mitochondrial metabolism, is primarily utilized by resting macrophages for efficient oxidative metabolism and ATP synthesis. However, upon activation by LPS, oxidative phosphorylation is suppressed and macrophages preferentially rely on glycolysis as an alternative mode of ATP production, despite its lower energy efficiency. Additionally, LPS triggers the generation of ROS from complex I in mitochondria through a mechanism that remains unidentified.^[^ ^40^ ^]^ In this study, we have demonstrated that RMVs possess the ability to modulate CI and CII activity, thereby stabilizing mitochondrial membrane potential affected by LPS and subsequently reducing both mitochondrial ROS and intracellular ROS production. Notably, 3‐HPA in RMVs plays a pivotal role. 3‐HPA has the potential to mitigate intracellular oxidative stress, decrease mitochondrial ROS production, and facilitate macrophage polarization toward an anti‐inflammatory phenotype. This effect is likely mediated through regulation of mitochondrial permeability. Specifically, LPS‐induced opening of the mitochondrial permeability transition pore initiates inflammation; however, 3‐HPA can inhibit this process and reverse LPS‐induced pro‐inflammatory macrophage transformation.

Bacterial signaling through vesicular forms offers several advantages, including but not limited to: 1) Protective effects: Vesicles can shield signaling molecules from external environmental influences, such as enzymatic degradation or attack by other cells, thereby ensuring the stability and efficacy of signaling molecules. 2) Long‐range transmission: Vesicles can disseminate through body fluids or intercellular space, enabling signaling molecules to affect distant cells or tissues and expand the scope of signal transmission. 3) Selective delivery: Vesicles can selectively load specific signaling molecules for precise signal delivery to cells or tissues while avoiding unnecessary effects on other cells. 4) Regulation of signal strength: By regulating the amount and frequency of vesicle release, bacteria can flexibly adjust the concentration and delivery rate of signaling molecules to adapt to different environmental conditions or growth stages.^[^ ^41^ ^]^ In recent years, numerous studies have substantiated the beneficial impact of EVs on wound healing. For instance, exosomes derived from cultured mesenchymal stem cells exhibit a remarkable ability to selectively target endothelial cells and stimulate angiogenesis.^[^ ^42^ ^]^ In contrast to EVs derived from human cells, the production of BEVs in large quantities is comparatively more feasible. In terms of efficacy, although RMV encompasses a diverse range of bioactive components, the effectiveness observed with 3‐HPA alone was comparable to that of RMV, suggesting a significant role played by 3‐HPA in treatment. Notably, the number of hair follicles in the skin of mice treated with RMVs was significantly higher compared to that in the 3‐HPA treatment group, indicating potential presence of additional constituents within RMVs facilitating skin structure recovery. Therefore, further investigation is warranted to elucidate the specific contributions of other constituents within RMV toward its overall efficacy.

Overall, RMVs expedited the process of oral mucosal wound healing, with macrophages likely serving as the target cells for RMVs in vivo. RMVs in in vitro studies promoted the shift of macrophages toward the M2 phenotype, possibly through the regulation of mitochondrial permeability and decrease in oxidative stress by 3‐HPA. Our discoveries offer fresh perspectives on how commensal MVs and 3‐HPA contribute to enhancing wound healing by influencing macrophage polarization. However, the targets and specific molecular pathways of RMVs affecting mitochondrial permeability remain unclear. Therefore, further investigations are warranted to elucidate the precise mechanisms through which RMVs impact mitochondrial function, in order to gain a deeper comprehension of the interplay between microorganisms and host immune cells.

## 4 Experimental Section

### Bacterial Strain

*L. reuteri* DSM 20 016 was purchased from the Genetimes Technology, Inc. (Shanghai, China). The bacteria were spread on MRS Agar and grown at 37 °C in an oxygen‐free environment with low levels of oxygen and carbon dioxide (O2 < 0.1% and 7% < CO2 < 15%) (Anaerogen Pak Jar, Oxoid Ltd).

### Basic Characterization of the Biofilms

The bacteria were inoculated into 90 mm diameter Petri dishes and incubated at 37 °C in an anaerobic environment for 24 h without agitation. Following incubation, non‐adherent cells were harvested, and the biofilms were washed with calcium and magnesium Phosphate Buffered Saline free (PBS; pH 7.2). After 24 h of incubation, the biofilms and their planktonic counterparts were centrifuged at 4000 × g for 20 min at 4 °C, then rinsed twice with PBS before being placed on glass coverslips (12 mm in diameter). The samples were analyzed using a scanning electron microscope (SEM; Hitachi, Japan).

### Isolation and Characterization of RMVs

The strains were grown in MRS medium at 37 °C with agitation (120 rpm rotation from the beginning of cultivation; the volume size of flask/the volume of culture = 5:1), harvested after 48 h, separated from the culture broth through centrifugation at 5000 × g for 20 min at 4 °C, and filtering residual cells from the supernatants using a 0.45 µm pore filter. Following this, the liquid above the sediment was spun in a Beckman Coulter Optima L‐80XP ultracentrifuge (Beckman Coulter, United States) at 129000 × g at 4 °C for 1.5 h. The liquid was removed, the solid particles mixed in PBS buffer, and then spun again at high speed (129000 × g at 4 °C for 16 h). The pellets were finally suspended in PBS, aliquoted and stored at −80 °C. RMVs morphology was examined using a transmission electron microscope (TEM; Hitachi, Japan) while the size distribution was determined through dynamic light scattering (DLS; Anton Paar, Austria), following previously detailed procedures.

### Mice

Eight‐week‐old female C57BL/6 mice were acquired from the Animal Center of Fourth Military Medical University (Xi'an, China). The mice were housed in a controlled environment free of specific pathogens, following a 12 h light and 12 h dark schedule. Every mouse was allowed to freely eat and drink. All animal experiments were approved by the Institutional Animal Care and Use Committee of the Fourth Military Medical University (kq‐2019‐079).

### Oral Ulcer Treatment in Mouse Models

Mice were administered pentobarbitone sodium (40 mg kg^−1^) through an intraperitoneal injection to be anesthetized. Creating an ulcer model involved placing a 3 mm circular filter paper soaked in 100% acetic acid on the mouse's tongue for 30 s, resulting in a circular ulcer within 24 h. Following the development of oral ulcers, the experimental group applied 10 µL of 200 µg mL^−1^ RMVs over the ulcer twice daily. The control group received an equivalent volume of saline solution. Every group contained 6 mice, and the weight of each mouse was measured daily. On day 7, The mouth sores were photographed and then euthanized all the mice to collect their tongues for additional examination.

### Cutaneous Wound Healing Model

A 10‐mm circular wound on the full‐thickness of the back was created after shaving and cleaning the area in a sterile manner. Four groups were formed with 6 mice in each group, and they were treated locally with PBS (PBS), hydrogel (Gel), hydrogel with 200 µg RMVs (Gel+RMV), hydrogel with 50 µg 3‐HPA (Gel+3‐HPA). Following the surgery, the wound was uniformly dressed with surgical bandages (3 m, 9546) and then removed. The wound sizes were photographed and measured with Image J at the indicated time points (days 0, 3, 7, 10, and 14). Mice were euthanized at 3, 7, and 14 days after the surgery, and the damaged regions were harvested for additional examination.

### Histological Analysis

The tongue and skin were then embedded in paraffin and 4 µm thick sections were obtained. After deparaffinization, the sections were stained with H&E and Masson according to the manufacturer's instructions. The slides were examined with a light microscope and pictures were captured with a stereomicroscope (Leica, Germany).

### Cell Culture

RAW 264.7 cells (Beyotime, C7505) were grown in high‐glucose Dulbecco's modified eagle medium (DMEM; Gibco), supplemented with 10% fetal bovine serum (FBS; Gibco). The cultures were kept in a humid environment with 5% CO_2_ at 37 °C.

### Immunofluorescence Analysis

The glass coverslips of macrophage were fixed with 4% paraformaldehyde at room temperature for 15 min. The tongue and skin samples were immersed in the ideal temperature solution (Leica, United States), resulting in 10 µm thin slices. The coverslips and the sections were permeabilized with 0.1% Triton X‐100 (Sigma‐Aldrich, 93 443) for 10 min and subsequently blocked using 5% BSA at 37 °C for 1 h. For macrophage analysis, the coverslips and the sections were incubated with Alexa Fluor 488‐anti‐mannose receptor (Abcam, ab195191) and Alexa Fluor 647‐anti‐iNOS (Abcam, ab209027) antibodies overnight at 4 °C. Hoechst 33 342 (Sigma‐Aldrich, 14 533) was used for nuclear counterstaining at room temperature for 10 min. Images were captured using the confocal microscope (Nikon, Japan) and the super‐resolution Live Cell Imaging microscope (ZEISS Elyra 7, Japan).

### ELISA

Fresh skin wound samples were collected and then mixed in RIPA buffer with a protease inhibitor while being kept cold on ice. Next, the liquid above the sediment was gathered following a spin at 12 000 rpm for 10 min at 4 °C. Protein concentrations in the liquid layer above skin wounds and in macrophage cultures were measured with ELISA kits for mouse IL‐10, TNF‐𝛼, IL‐6, IL‐1β, MDA, and NO (MultiSciences, China). Every operation was carried out in compliance with the guidelines provided by the manufacturer.

### Western Blot Analysis

Cells were lysed on ice using radio‐immunoprecipitation assay (RIPA) buffer (Beyotime, China) containing protease inhibitor (Roche, 0 469 313 2001). Protein levels was measured using the BCA protein assay reagent (Beyotime, China). Twenty micrograms of protein underwent separation using sodium dodecyl sulfate (SDS) polyacrylamide gel electrophoresis and were then transferred to polyvinylidene fluoride (PVDF) membranes (Millipore, Germany). Following a 1 h block in 5% BSA (MP Biomedicals) at room temperature, the membranes were then exposed to primary antibodies against iNOS (Cell Signaling Technology, 13120S), Arginase‐1 (Cell Signaling Technology, 93668S), and 𝛽‐tubulin (CWBIO, CW0098) overnight at 4 °C. After being washed with tris buffered saline tween (TBST), the membranes were then incubated with the necessary secondary antibodies for 1 h at room temperature. The blots were ultimately observed using a Western‐Light Chemiluminescent Detection System (Tanon‐Bio, China).

### Flow Cytometry

For the examination of surface markers, cells were labeled with FITC‐conjugated anti‐CD80 (BioLegend, 101 206), PE‐conjugated anti‐CD86 (BioLegend, 127 607) and FITC‐conjugated anti‐CD206 antibodies (BioLegend, 128 005) for 30 min on ice in the absence of light. After being washed twice, the cells were then analyzed using a flow cytometer (Cytomics FC 500, Beckman‐Coulter).

### Real‐Time PCR

The total RNA was extracted with TRIzol LS (Life Technologies, United States) and measured with a Nanodrop 2000 UV–visible spectrophotometer. Total RNA ranging from 20 to 100 ng ml^−1^ was utilized to create cDNA through a high capacity cDNA reverse transcription kit (Analytik Jena, United States), following the provided guidelines. SYBR Green probes specific for IL‐10, TNF‐α, IL‐1β, and IL‐6 were used in real‐time qPCR analysis of cDNA. Information regarding the primers utilized is available in Table S4 (Supporting Information). The C1000 Touch PCR System (Bio‐Rad, United States) qPCR was used for qPCR analysis.

### ROS Measurement

Cells were plated at 0.5 × 10^6^ cells mL^−1^ in black 96‐well plates (100 µL per well) and treating them as needed. ROS levels were measured with a bioluminescent kit (Beyotime, China) for ROS quantification, following the provided guidelines.

### Mitochondrial Complex Activity Measurement

Cells were plated at 0.5 × 10^6^ cells mL^−1^ in 10 cm non‐cell culture‐treated dishes (10 mL per dish) and treating them as needed. Mitochondrial complex I and II activity were measured with the CheKine Micro Mitochondrial complex I Activity Assay Kit and CheKine Micro Mitochondrial complex II Activity Assay Kit (Abbkin, China) following the provided guidelines.

### Mitochondrial Permeability Measurement

Cells were plated at 0.5 × 10^6^ cells mL^−1^ in confocal dish (1 mL per dish) and treating them as needed. Mitochondrial permeability was measured with the MPTP Assay Kit (Beyotime, China) following the provided guidelines.

### Statistical Analysis

All data were expressed as the means ± standard deviation (SD). Statistical comparisons between datasets were performed by analyzing normality and variance. Statistical significance between two groups was calculated by Student's t‐tests (two‐tailed). One‐way analysis of variance (ANOVA) was used to compare differences among multiple groups, and Tukey's post hoc test was used for multiple post hoc comparisons to determine the significance of differences between the groups after one‐way ANOVA. The Kruskal–Wallis H‐test or Mann–Whitney U‐test was used if the data did not follow a normal distribution. P values less than 0.05 were considered statistically significant. Statistical analysis was performed using SPSS software (IBM, 19.0).

## Conflict of Interest

The authors declare no conflict of interest.

## Author Contributions

Y.C., X.H., and A.L. contributed equally to this work. Y.C. and A.L. contributed to the study design, data collection, and paper preparation. X.H. undertook a substantial amount of work during the supplementary experiment phase. S.F. and M.L. contributed to the RMVs preparation. X.H., S.F., M.W., S.L., and Z.L. contributed to the in vitro experiments. X.Y., P.L., H.G., and F.F. contributed to the animal study. S.L. and K.X. developed the concept, supervised the research, and critically revised the paper. All authors contributed to the paper and approved the final paper.

## Supporting information

Supporting Information

## Data Availability

The data that support the findings of this study are available from the corresponding author upon reasonable request.
