# Supplementary material for: Lactobacillus Reuteri Vesicles Regulate Mitochondrial Function of Macrophages to Promote Mucosal and Cutaneous Wound Healing
Source: Adv Sci (Weinh). 2024 Apr 22;11(24):2309725. doi: 10.1002/advs.202309725 (PMC11199966; doi:10.1002/advs.202309725)
Supplement: Supplementary file 1 — Supporting Information [file ADVS-11-2309725-s001.pdf]

ADVANCED SCIENCE
Open Access

Supporting Information

for *Adv. Sci.*, DOI 10.1002/advs.202309725

Lactobacillus Reuteri Vesicles Regulate Mitochondrial Function of Macrophages to Promote Mucosal and Cutaneous Wound Healing

Yuan Chen, Xiaoyao Huang, Anqi Liu, Siyuan Fan, Shiyu Liu, Zihan Li, Xiaoxue Yang, Hao Guo, Meiling Wu, Meng Liu, Peisheng Liu, Fei Fu, Siying Liu and Kun Xuan**

Supporting Information

Lactobacillus reuteri vesicles regulate mitochondrial function of macrophages to promote mucosal and cutaneous wound healing

Yuan Chen, Xiaoyao Huang, Anqi Liu, Siyuan Fan, Shiyu Liu, Zihan Li, Xiaoxue Yang, Hao Guo, Meiling Wu, Meng Liu, Peisheng Liu, Fei Fu, Siying Liu,* and Kun Xuan*

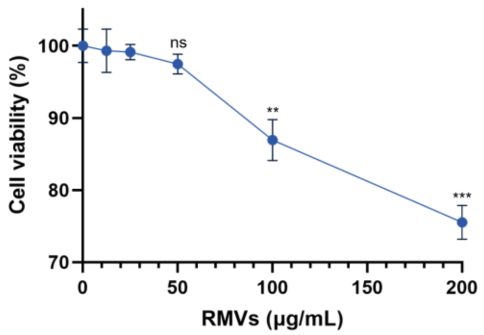

Figure S1. Impact of varying concentrations of RMVs on macrophage activity

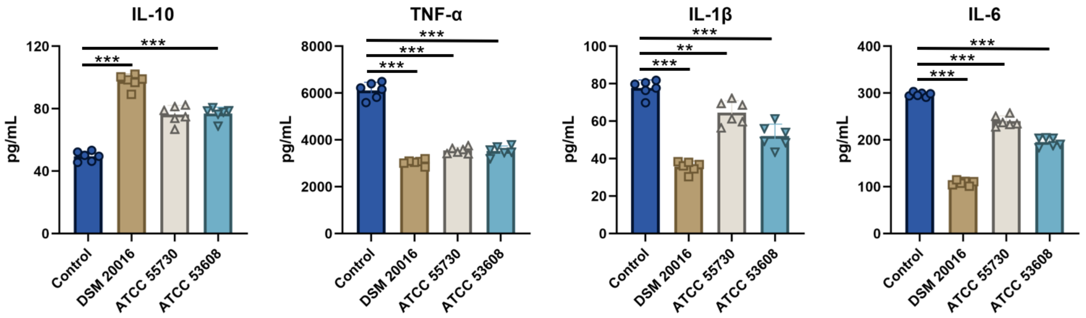

Figure S2. Repercussions of vesicles derived from distinct strains of *Lactobacillus reuteri* on macrophage cytokine secretion.

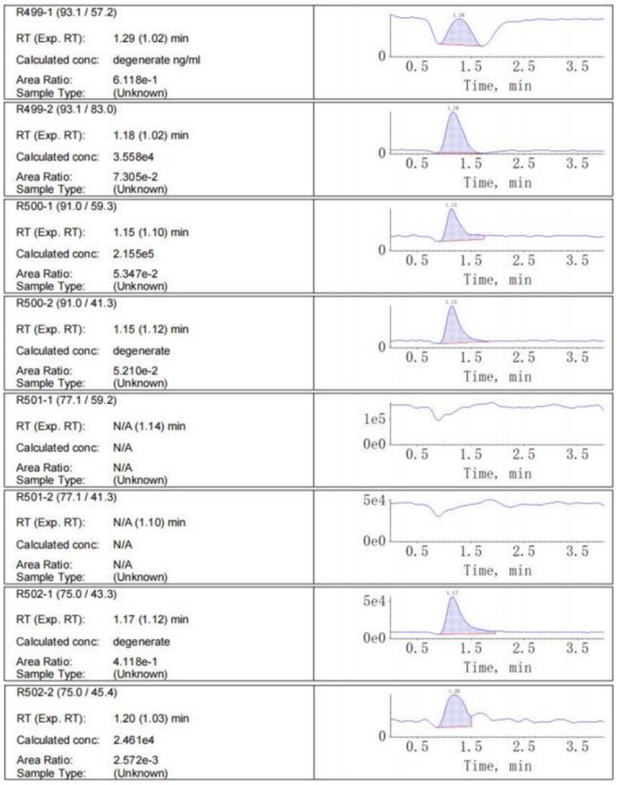

Figure S3. Total ions current of RMVs

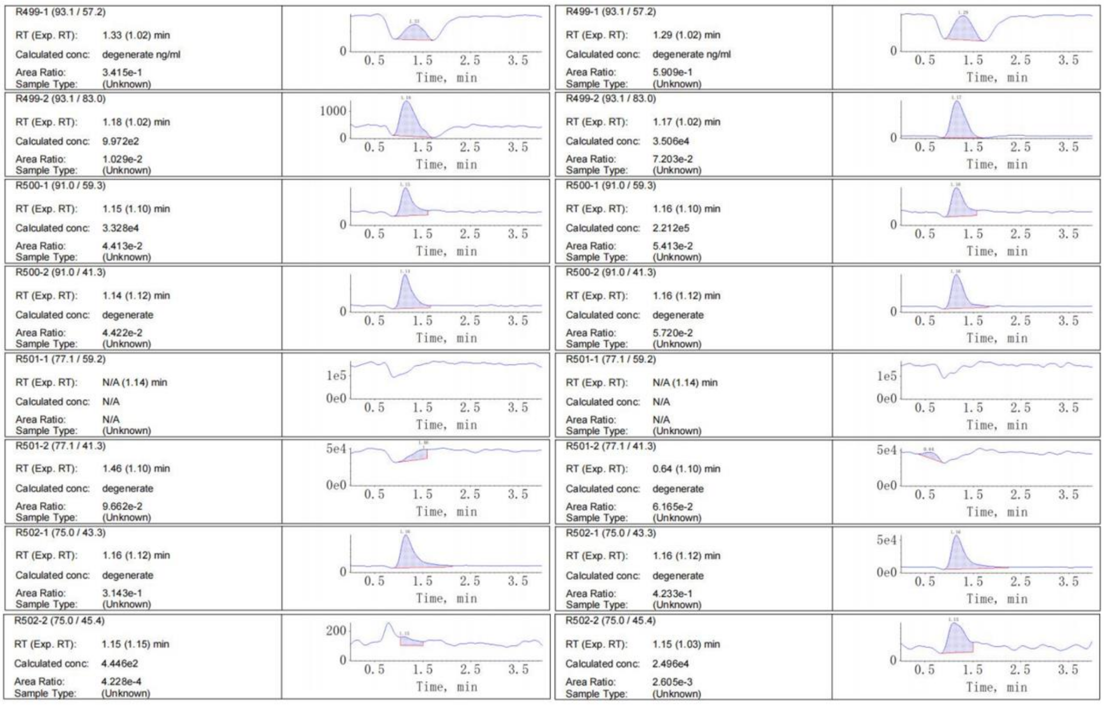

Figure S4. Total ions current of macrophages

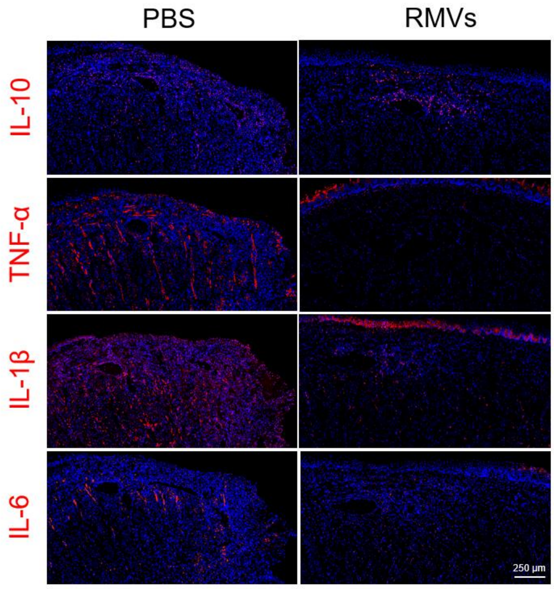

Figure S5. Representative immunofluorescence staining results of inflammatory factors in the lingual mucosa

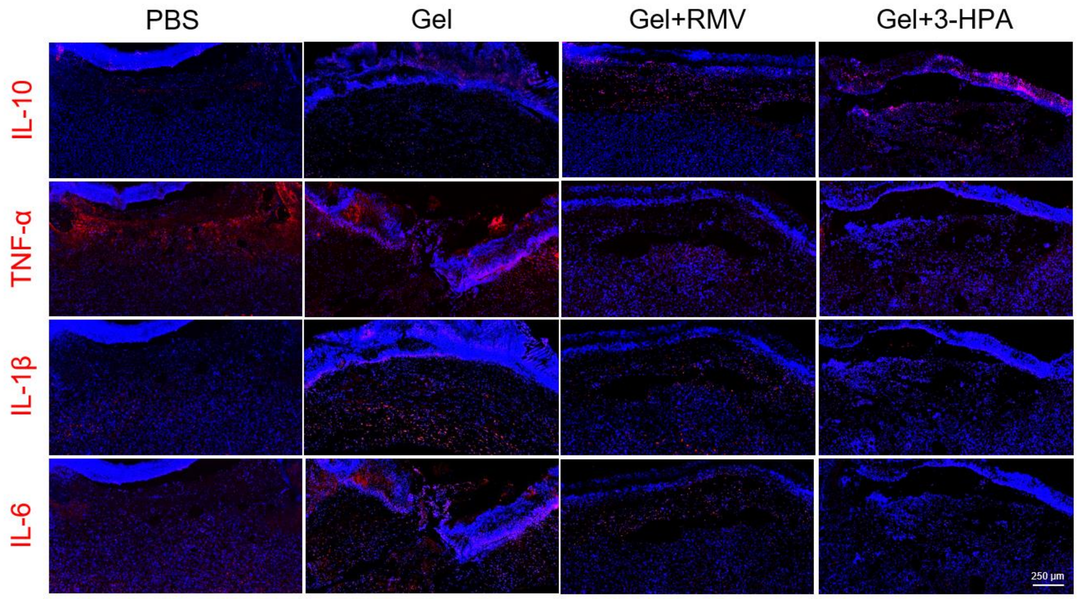

Figure S6. Representative immunofluorescence staining results of inflammatory factors in skin

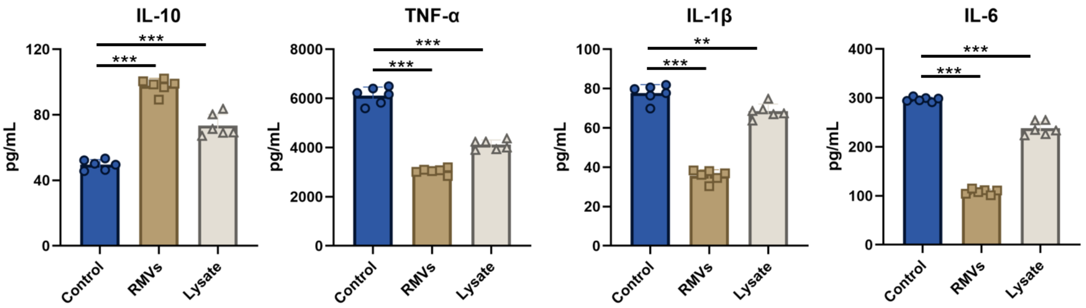

Figure S7. Repercussions between RMVs and *L. reuteri* lysate on macrophage cytokine secretion

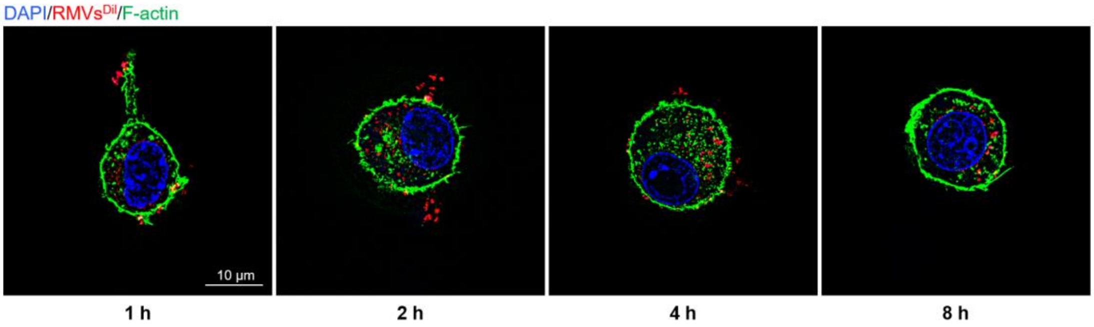

Figure S8. Images of RMVs captured by macrophages

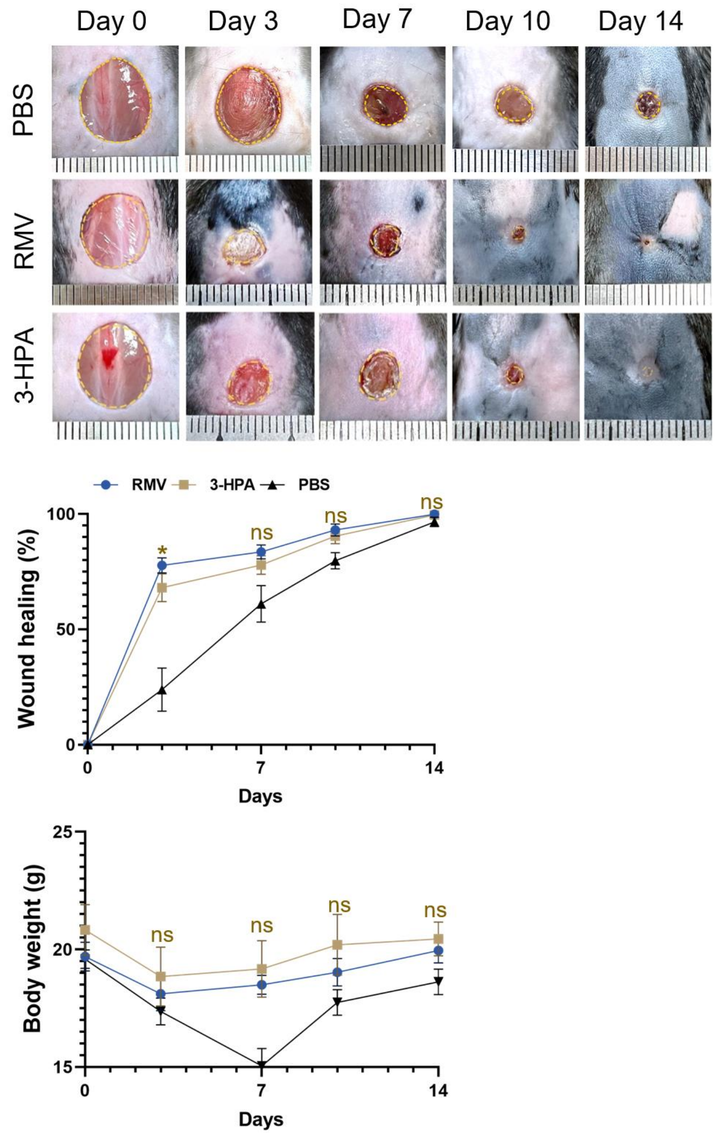

Figure S9. Comparison between 3-HPA and RMVs in their efficacy for promoting skin wound healing.

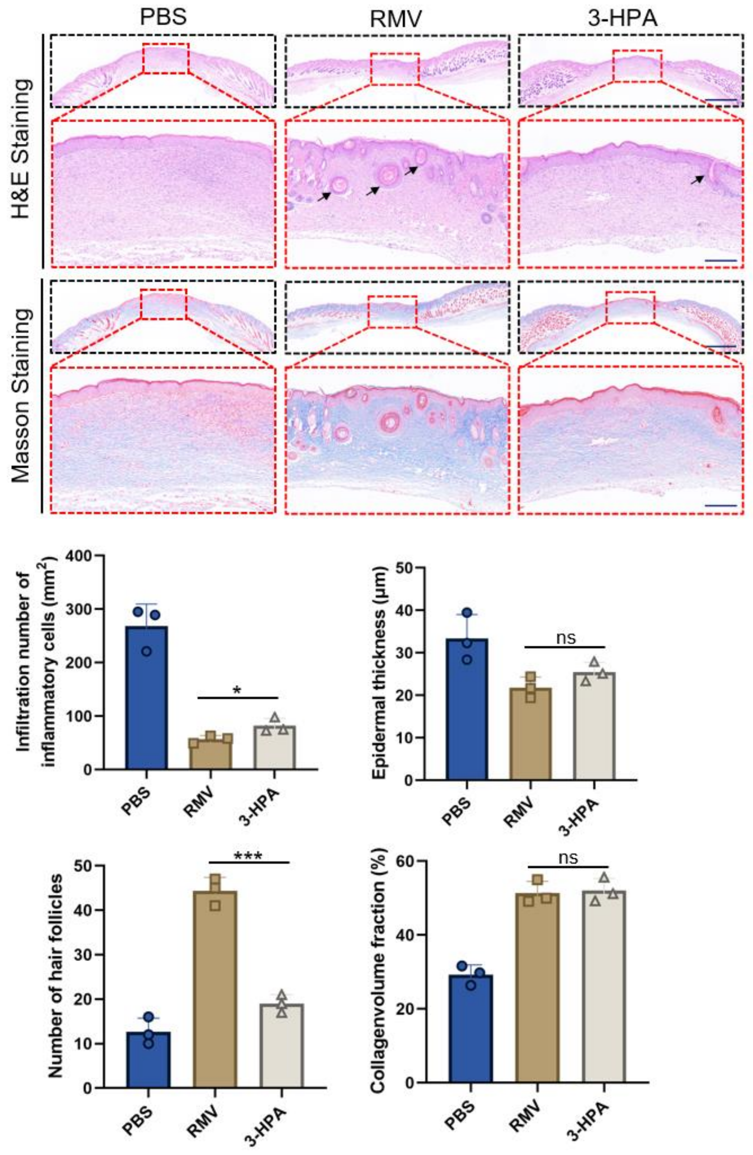

Figure S10. Comparison of wound histological healing after 3-HPA and RMVs treatment

ng/mL	3-Hydroxy propionaldehyde	Glycerol	Trimethylene glycol	β-Hydroxy propionic acid
RMVs	24781.89	35318.74	N.D.	218375.48

Table S1. Quantification of 3-HPA in RMVs

ng/mL	3-Hydroxypropionaldehyde	Glycerol	Trimethylene glycol	β-Hydroxy propionic acid
Before	N.D.	1348.05	N.D.	25689.44
After	448.55	986.11	N.D.	32312.12

Table S2. Quantification of 3-HPA in macrophages before and after the addition of RMVs

Primer names	Sequence (5' to 3')
m-IL-10-Forward	CTTACTGACTGGCATGAGGATCA
m-IL-10-Reverse	GCAGCTCTAGGAGCATGTGG
m-TNF-α- Forward	CAGGCGGTGCCTATGTCTC
m-TNF-α-Reverse	CGATCACCCCGAAGTTCAGTAG

m-IL-1β- Forward	GAAATGCCACCTTTTGACAGTG
m-IL-1β- Reverse	TGGATGCTCTCATCAGGACAG
m-IL-6- Forward	CTGCAAGAGACTTCCATCCAG
m-IL-6- Reverse	AGTGGTATAGACAGGTCTGTTGG

Table S3. Primer sequences for qRT-PCR

No.	Volume (μL)	Protein concentration (μg/mL)	Particle concentration (particles/ mL)	Mean grain size (nm)
1	900	51.6	5.50E+10	99.8
2	900	55.6	5.80E+10	105.0
3	900	55.2	4.70E+10	105.1

Table S4. Analysis of RMVs concentration

空军军医大学口腔医院实验动物伦理审查表（仅供课题申报）

Application for Laboratory Animal Welfare and Ethical review , School of Stomatology, Air Force Military Medical University(Used only for Application)

受理编号 Appl. No: kq-2021-079

<table>
<tr><td rowspan="4">项目与人员信息
Study Information and Applicant Information</td><td colspan="2">课题名称 Program ：罗伊氏乳杆菌囊泡调节巨噬细胞线粒体功能促进黏膜与皮肤伤口愈合</td></tr>
<tr><td>申请人 Aapplicant ：陈媛</td><td>科室 Department：口腔预防科</td></tr>
<tr><td>学历 Education：本科</td><td>技术职称 Professional title： ■无</td></tr>
<tr><td>联系电话 Telephone：17691218706</td><td>邮箱 Email：1054101990@倾 qq.com</td></tr>
<tr><td rowspan="4">动物信息
Animal to be used</td><td>动物来源单位 Source of animal：空军军医大学动物中心</td><td>来源单位生产许可证编号 Certificate number：SCXK(陕）2019-001</td></tr>
<tr><td>品种品系 Species or strain：C57BL/6J 小鼠</td><td>等级 Grade：■普通级 □SPF 级</td></tr>
<tr><td>规格 Specifications：8 周</td><td>数量 Number：48 只（雌 48 只；雄 0 只）</td></tr>
<tr><td colspan="2">饲养环境 Condition of the Housing Facility：■普通环境 □屏障环境</td></tr>
<tr><td rowspan="3">实验内容
Outline of experiments</td><td colspan="2">实验目的 Aim of experiment:
(1) 建立小鼠舌黏膜溃疡模型与皮肤缺损模型；
(2) 验证罗伊氏乳杆菌囊泡对小鼠舌黏膜溃疡及皮肤缺损愈合的促进作用。</td></tr>
<tr><td colspan="2">实验方法 experimental methods:（①动物分组，每组动物数量。②涉及到药品请写明给药方式、药品名称、使用浓度、剂量和频次。③动物标识。④动物保定。）
① 动物分组：分为 6 组，舌对照组、舌 RMVs 组、皮肤对照组、皮肤凝胶组、皮肤 RMVs 凝胶组、皮肤 3-HPA 凝胶组（每组 8 只）；
② 药品：腹腔注射 1 %戊巴比妥钠 0.1 mL + 局部注射 1 %利多卡因 0.02 mL；
③ 动物标识：耳标标记法；
④ 动物保定：以一手抓住大鼠尾根部，另一手拇指和食指捏住耳后颈部皮肤，其余三指和掌心相对抓住前背部的皮肤，可控制小鼠的头部和前肢。</td></tr>
<tr><td colspan="2">主要观察指标 Main observation target:
1. 组织学染色检查：对处理后的组织进行 H&E 和 Masson 染色观察舌黏膜溃疡及皮肤缺损部位的炎症细胞浸润程度、新形成组织的形态；
2. 免疫荧光染色：观察巨噬细胞表型比例；
3. ELISA 检测：检测炎症因子水平变化。</td></tr>
</table>

<table>
<tr><td rowspan="4">动物福利
Animal
welfare</td><td colspan="4">造成动物不适或疼痛的情况及相应处理方法 Discomfort or pain caused by the experiment, please describe the treatment:
1、疼痛：■短暂或轻微。☐可通过适当方法缓解。☐持续而无法缓解。☐强烈而无法缓解。

2、处理方法（根据疼痛类型选择，可多选）：
■优化实验操作，加强操作人员培训，减少实验过程中的不适。
■控制饲养密度，增加环境丰富度。
☐根据情况，在手术前给予镇静剂或止痛药。
☐根据情况，尽早选择仁慈终点。
☐其他，请说明：</td></tr>
<tr><td colspan="4">术后护理 postoperative care:
1、手术类型：☐不进行外科手术 ☐终末性手术（术后不要求动物复苏，不进行术后护理。）
■存活性手术（术后要求动物复苏，需进行术后护理。）

2、存活性手术术后护理（可多选）：
■术后进行保温，加强营养，加速动物恢复。
☐术后使用抗生素预防感染，必要时使用镇痛剂。
☐其他，请说明：</td></tr>
<tr><td colspan="4">动物处死方法 executing animal method ：■过量麻药 ☐颈椎脱臼 ☐CO_2吸入
☐其他：</td></tr>
<tr><td colspan="4">是否使用有毒（害）物质（感染、放射、化学毒、其他）Poisonous（harmful） material（infection, radiate, chemical poison and other）being used:
☐是 yes 说明 Declare: ■否 no</td></tr>
<tr><td>声明
Declaration</td><td colspan="4">1、我将自觉遵守实验动物福利伦理相关法规和各项规定，同意接受伦理委员会和实验动物室管理者的监督与检查（I will abide by the law and regulation stipulation, and accept the supervision and inspection by the committee and laboratory animal department..）。
2、本人保证本申请表中所填内容真实、详尽和易懂（The information I have given is accurate, detailed and comprehensive.）。

申请人签名 Signature of applicant: 陈[illegible]

申请日期 Application date: 2021年Y 9月M 10日D</td></tr>
<tr><td>福利伦理委员会审批意见
Approval opinion of Committee</td><td>主任委员签（章）
Signature (stamp) of Chairman of Committee:
[illegible]</td><td>同意 Agree☑</td><td>不同意 Disagree☐</td><td>签章 Stamp</td></tr>
</table>

备注：1、正反双面打印，如果表格变成 3 页，请调整。

2、该审查表仅用于课题申请时使用，编号为受理编号，不涉及实验的实际开展及过程监督，不能用于文章发表、项目结题。正式开展动物实验时，请重新填写动物伦理审查表，获得批准编号。

Table S5. Animal Ethics Review Form
